# Efficient regeneration system from rye leaf base segments

**DOI:** 10.1186/s40064-016-3689-9

**Published:** 2016-11-24

**Authors:** Kamil Haliloglu, Murat Aydin

**Affiliations:** Department of Field Crops, Faculty of Agriculture, Atatürk University, 25240 Erzurum, Turkey

**Keywords:** Plant regeneration, 2,4-D, TDZ, Rye

## Abstract

Rye is second only to wheat among grains most widely used in the making of bread and is also a very important gene resource for breeding and improvement of wheat and other cereal crops owing to tolerance to abiotic stress factors such as low temperatures, drought and poor soil conditions. However, application of biotechnologies has been limited in rye breeding since it is one of the most recalcitrant species in tissue culture. A simple and fast regeneration system from leaf-base segment explant of rye was developed in this study. Basal media, carbohydrate source, combination of plant growth regulators and the leaf segment locations were evaluated for callus and shoot formation. The highest callus formation (10.39%) and shoot formation (4.53%) were achieved from first basal segments 3–4 days old seedlings. MS (Murashige and Skoog, in Physiol Plant 15:473–497, 1962) medium supplemented with 30 g/L sucrose and 2 mg/L 2,4-D (2-4 dichlorophenoxyacetic acid) + 1 mg/L TDZ (Thidiazuran) was the best medium for shoot formation (18.75%) in first leaf base segment culture. Regenerated plants were phenotypically normal and set seed after they were successfully transferred to soil. The results indicate that this regeneration method can be used for genetic transformation in rye.

## Background

Rye (*Secale cereale* L.), is an annual, diploid and cross-pollinated crop. It is also an important crop in Turkey and widely distributed across the world. Its importance is due to resistance against good winter hardiness, drought and the ability to produce a crop on acid and sandy soils which is not suitable for other cereal crops. Rye also forms a very important gene resource for breeding of wheat and other cereal crops. However, rye is well known as recalcitrant cereal crop in tissue culture and remains to be a challenging task for plant biotechnology. With the use of new technologies in molecular biology and genetic engineering, plant transformation has become leading fundamental issue in plant molecular breeding. Primary condition for the effective use of biotechnology in crop breeding is to have efficient in vitro plant regeneration system from cultured cells and tissues. Early studies of rye somatic embryogenesis and in vitro plant regeneration from various explant sources of young leaf segment (Linacero and Vazquez 1986), young inflorescence (Krumbiegel-Schroeren et al. 1984; Linacero and Vazquez 1990; Rakoczy-Trojanowska and Malepszy 1993) and root organ cultures (Whitney 1996) have been described in several articles. However, the low somatic embryo formation and subsequently plant regeneration from somatic embryos are problematic. The most effective tissue source for regenerating whole plants has been reported as immature embryos (Popelka and Altpeter 2001; Rakoczy-Trojanowska and Malepszy 1993; Zimny and Lorz 1989) and are used comprehensively in genetic transformation studies of rye (Popelka and Altpeter 2003).

Growth conditions of donor plants influence considerably in vitro regeneration of immature embryos (Maes et al. 1996; Vasil et al. 1993). Furthermore, growing donor plants for immature embryo culture is labor intensive, time consuming and costly. Advantages of leaf segments in in vitro generation system can be listed as the most easily available donor material which can be grown in vitro and a short-term, frequent source of explants can be supplied (Haliloglu 2006). The aim of this study was to improve a repeatable, reliable and simple in vitro regeneration system from rye leaf base segments. In this study, basal media, carbohydrate source, plant growth regulator combinations and orientation of leaf segment on callus and plant regeneration from leaf bases were examined. Our results are expected to be helpful as an identified regeneration system for genetic transformation studies.

## Methods

Mature seeds of diploid rye genotype (*Secale cereale* L.) obtained from Atatürk University, Faculty of Agriculture, Department of Field Crops were used as plant material. The seeds were surface-sterilized with 70% ethanol for 5 min and with sodium hypochlorite for 15 min and then rinsed with sterile water. The seeds were cultured under light on moist filter paper in Petri dishes for germination. Two leaf segments from leaf base to tip (referred as 1–2) and each 2–3 mm in length were taken from leaf base of 3- to 4-day old seedlings. Leaf segments were cultured on the callus initiation medium at 25 ± 1 °C in the dark, and 30 day later, the callus induction for each segment were measured. Callus formation was periodically observed. MS medium and N6 (Chu 1975) were compared for callus formation and growth. For callus induction, media with varying concentrations of different plant growth regulators (A. 1 mg/L 2,4-D + 1 mg/L TDZ, B. 1 mg/L 2,4-D + 2 mg/L TDZ, C. 2 mg/L 2,4-D + 1 mg/L TDZ and D. 2 mg/L 2,4-D + 2 mg/L TDZ) and different carbon hydrate source (30 g/L sucrose and 40 g/L maltose) were tested. The media were solidified with Phytagel (2 g/L) and the pH was adjusted to 5.8 with NaOH prior to autoclaving.

For shoot regeneration, callus was transferred onto regeneration medium. Regeneration medium was the same as callus initiation medium which was either MS or N6 medium supplemented with either 30 g/L sucrose and 40 g/L maltose based on experiment without any plant growth regulator. They were cultured under 16-h photoperiod (62 μmol m^−2^ s^−1^) at 25 ± 1 °C. Number of shoots (1.5 cm in length or longer) were recorded at 30 days after transferring to regeneration media. Regenerated plants having well-developed roots were transferred to soil. After 30 d in callus induction medium, callus formation was measured. The callus formation percentage formulated as (number of segments forming callus/number of cultured segments) × 100. The shoots formation percentage was calculated by division of total number of regenerated plantlets over the number of cultured segments. Experiment was conducted in completely randomized design (CRD) with 4 replications of 25 segments per Petri dishes. Data were analyzed by ANOVA (basal media × carbohydrate source × combination of plant growth regulators; 2 × 2 × 4) using SAS/PC statistical program (SAS Institute Inc., Cary). Fisher’s Least Significant Difference (LSD) was used to compare means by using Test at 0.05 level.

## Results

In order to have successful in vitro culture of cells and tissues, factors such as explant type and culture conditions are important. In this study, parameters affecting differentiation in cells of leaf segments were investigated.

### Effect of base segments on callus and shoot formation

**Fig. 1:**
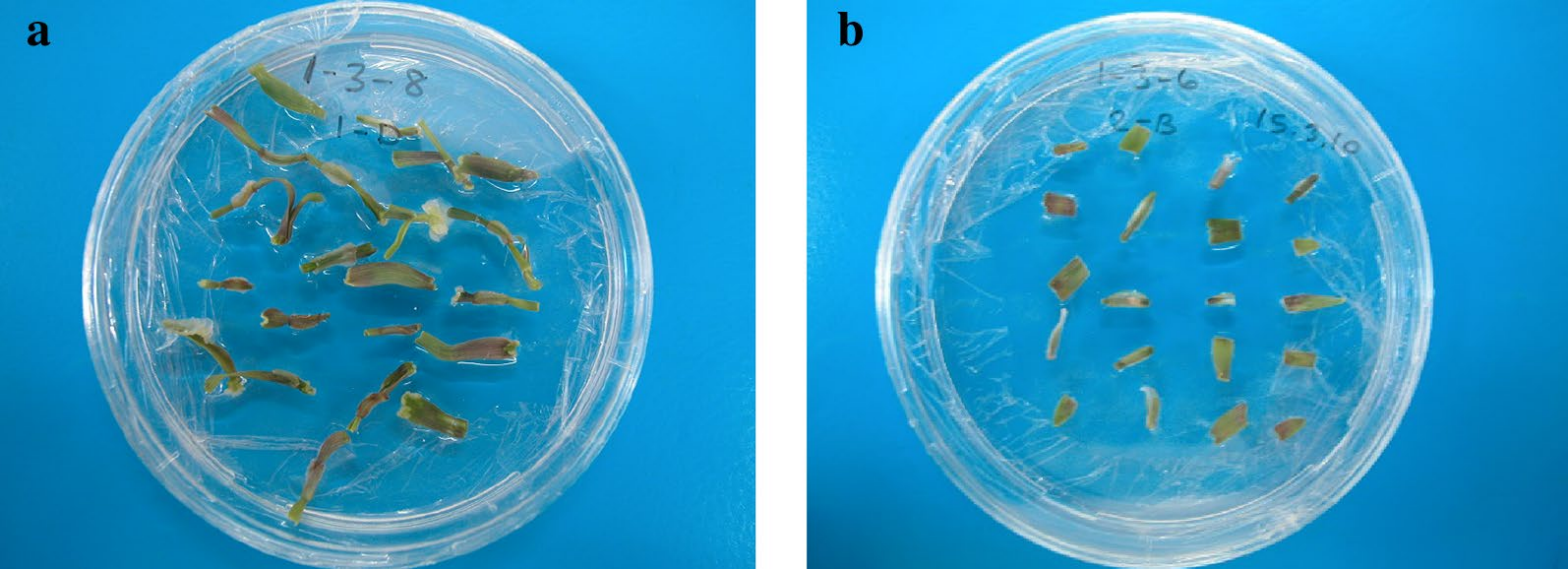
Responses of first (**a**) and second segments (**b**) of rye (*Secale cereale*) on MS medium supplemented with 1 mg/L 2,4-D + 2 mg/L TDZ plus 30 g/L sucrose for callus initiation and shoot formation

First segments responded well to in vitro culture conditions in terms of used plant nutrient medium, carbohydrate source and plant growth regulator combinations (Fig. 1). According to analysis of variance, there were significant differences among the location of leaf base segments in respect to callus formation (p < 0.01) as well as shoot formation (p < 0.01). The highest callus formation rate and shoot formation were observed in first segments. On the other hand, both callus and shoot formation in the second base leaf were close to zero (0.08%).

These results clearly indicate that leaf base segment location on the plant which was used as an explant has an important role in callus and shoot formation. When distance of explants to base meristem gets far, callus formation, somatic embryo formation and plant regeneration were decreased and lost their embryogenic capacity consequently. Growth and development of explant were observed only in the second leaf base segment. Later on, their colors turned to brown and dark color. In addition, no somatic embryo formation and plantlets regeneration were observed. Therefore, segment type had been removed from variance analysis and the data of first segment subjected to variance analysis and the results are given below.

### Effect of different basal medium on callus and shoot formation

Two different media type, N6 and MS medium were investigated. N6 and MS media consisted of different major and minor elements salts. Variance analysis showed that there were significant differences among media used in terms of callus formation (p < 0.01) and shoot formation (p < 0.01) (Table 1). The highest callus (15.78%) and shoot (6.72%) formation were observed in MS medium. On the other hand, certain decreases in callus formation and shoot formation were determined in N6 medium in which callus and shoot formation rate were 5.00 and 2.34%, respectively (Table 1). Shoot formation was influenced by basal medium × carbohydrate source and basal medium × combination of plant growth regulators interactions. Sucrose for MS basal medium (10%) and maltose for N6 basal medium (2.81%) was superior for shoot formation. In addition, 2 mg/L 2,4-D + 1 mg/L TDZ for MS medium (13.75%) and 2 mg/L 2,4-D + 2 mg/L TDZ for N6 medium (4.38%) gave better results for shoot formation. Overall, MS medium supplemented with 30 g/L sucrose and combination of 2 mg/L 2,4-D + 1 mg/L TDZ resulted in the highest shoot formation (18.75%).

**Table 1:** Callus and shoot formation (%) from base segments of rye (*Secale cereale*) on MS and N6 medium supplemented with carbohydrate and plant growth regulators

Basal medium	Carbohydrate source	Combination of plant growth regulators^a^	Callus formation (%)	Shoot formation (%)
MS	Maltose	A	12.50	2.50
		B	15.00	0.00
		C	27.50	8.75
		D	11.25	2.50
		Average	16.56	3.44
	Sucrose	A	16.25	3.75
		B	7.50	7.50
		C	15.00	18.75
		D	21.25	10.00
		Average	15.00	10.00
	Average	A	14.38	3.13
		B	11.25	3.75
		C	21.25	13.75
		D	16.25	6.25
		Average	15.78	6.72
N6	Maltose	A	1.25	3.75
		B	1.25	1.25
		C	12.50	3.75
		D	12.50	2.50
		Average	6.88	2.81
	Sucrose	A	2.50	0.00
		B	1.25	0.00
		C	1.25	1.25
		D	7.50	6.25
		Average	3.13	1.88
	Average	A	1.88	1.88
		B	1.25	0.63
		C	6.88	2.50
		D	10.00	4.38
		Average	5.00	2.34
The average of medium	Maltose	A	6.88	3.13
		B	8.13	0.63
		C	20.00	6.25
		D	11.88	2.50
		Average	11.72	3.13
	Sucrose	A	9.38	1.88
		B	4.38	3.75
		C	8.13	10.00
		D	14.38	8.13
		Average	9.06	5.94
	Average	A	8.13	2.50
		B	6.25	2.19
		C	14.06	8.13
		D	13.13	5.31
		Average	10.39	4.53
F value	Basal medium (M)		42.383**	13.364**
	Carbohydrate source (C)		2.573^ns^	5.523*
	Combination of plant growth regulators (R)		5.255**	5.386**
	M × C		0.436^ns^	9.818**
	M × R		1.125^ns^	3.773*
	C × R		4.234*	1.477^ns^
	M × C × R		2.050^ns^	0.682^ns^
LSD_(0.05)_	Basal medium (M)		3.33	2.41
	Carbohydrate source (C)		–	2.41
	Combination of plant growth regulators (R)		4.71	3.40
	M × C		–	3.40
	M × R		–	4.81
	C × R		6.66	–
	M × C × R		–	–

^a^A: 1 mg/L 2,4-D + 1 mg/L TDZ; B: 1 mg/L 2,4-D + 2 mg/L TDZ; C: 2 mg/L 2,4-D + 1 mg/L TDZ; D: 2 mg/L 2,4-D + 2 mg/L TDZ

* Significant at the 0.05 probability level

** Significant at the 0.01 probability level

^ns^ Non significant

### Different combination of plant growth regulators on callus and shoot formation

Analysis of variance indicated that there were significant differences among 4 different combinations of plant growth regulators for both callus (p < 0.01) and shoot formation (p < 0.01) (Table 1). The highest callus formation (14.06%) was observed in medium C containing 2 mg/L 2,4-D + 1 mg/L TDZ. It was followed by medium D which contain 2 mg/L 2,4-D + 2 mg/L TDZ with value of 13.13% (Table 1). They also fall into same group based on LSD Multiple Range test. Medium A (1 mg/L 2,4-D + 1 mg/L TDZ) and B (1 mg/L 2,4-D + 2 mg/L TDZ) produced 8.13 and 6.25% callus, respectively. Increases of 2,4-D concentration which is one of the auxin hormones in the combination induced callus formation. On the other hand, increases of TDZ (1 mg/L increment) concentration in combination decreases the callus formation (Table 1). In addition, interaction of carbohydrate source × combination of plant growth regulators influenced significantly callus formation. The highest callus formation in medium supplemented with maltose was observed medium C supplemented with 2 mg/L 2,4-D + 1 mg/L TDZ, while the highest callus formation resulted in medium D supplemented with 2 mg/L 2,4-D + 2 mg/L TDZ plus sucrose.

When four different combination of plant growth regulators were considered respect to shoot formation percentage, there were significant differences among combinations (p < 0.01). The highest shoot formation (8.13%) was observed in medium C containing 2 mg/L 2,4-D + 1 mg/L TDZ. It was followed by medium D (5.31%) with 2 mg/L 2,4-D + 2 mg/L TDZ (Table 1). The lowest (2.19%) shoot formation was obtained in medium B containing 1 mg/L 2,4-D + 1 mg/L TDZ (Table 1).

### Different carbohydrate sources on callus and shoot formation

Different carbohydrate sources such as, 30 g/L sucrose and 40 g/L maltose were assessed to determine the effect on callus and shoot formation. Based on analysis of variance, there were no significant differences between different carbohydrate sources in terms of callus formation. On the other hand, there was significant difference between carbohydrate sources with respect to shoot formation (p < 0.05). The highest callus formation rate (11.72%) was observed in medium containing 40 g/L maltose. Based on shoot formation, sucrose (5.94%) performed better than maltose (3.13%) (Table 1).

**Fig. 2:**
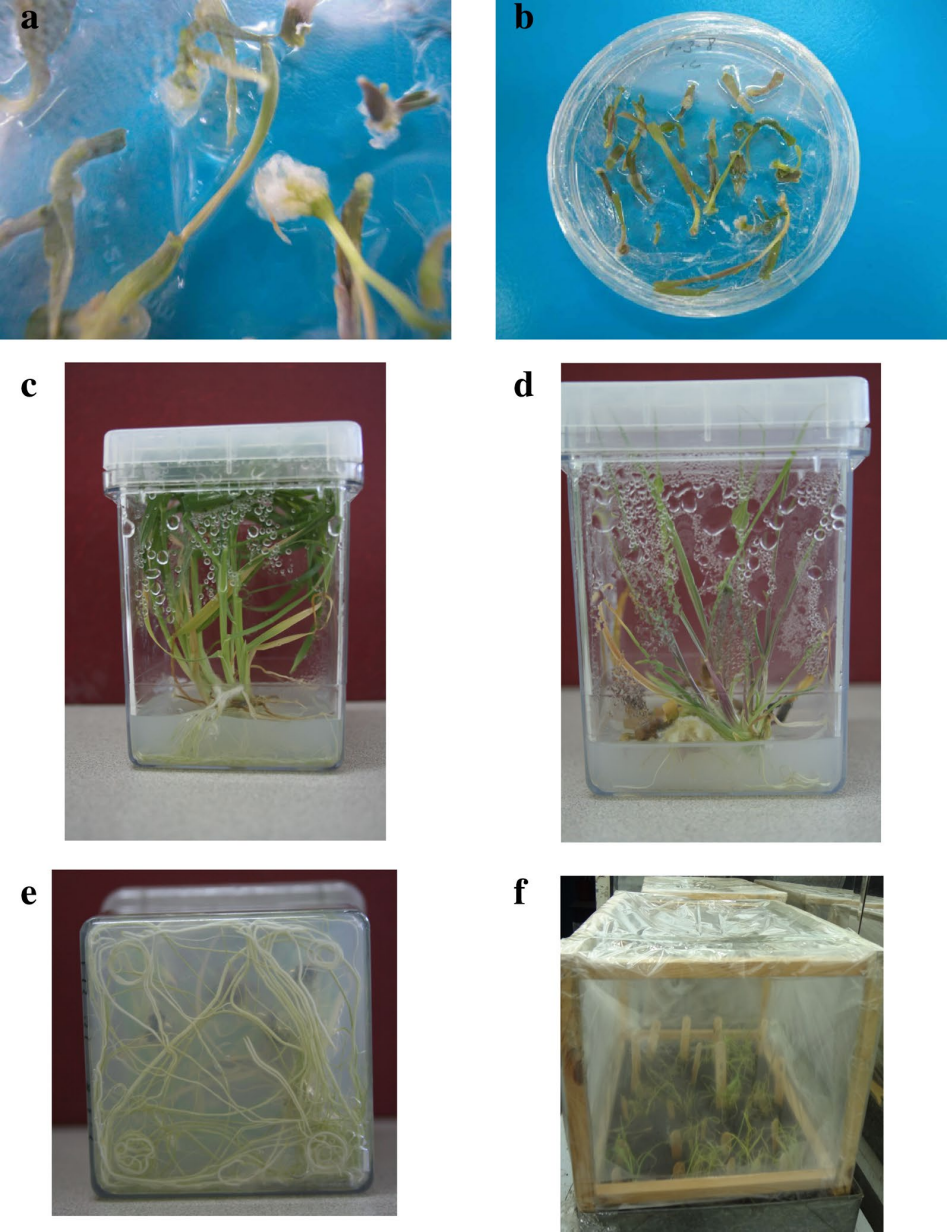
Developmental stages of first leaf base culture of rye. **a** Callus formation in MS medium supplemented with 1 mg/L 2,4-D + 1 mg/L TDZ plus 30 g/L sucrose, **b** Shoot formation in MS medium supplemented with 1 mg/L 2,4-D + 1 mg/L TDZ plus 30 g/L sucrose, **c** Plantlets with root and shoot in MS medium supplemented with 1 mg/L 2,4-D + 1 mg/L TDZ plus 30 g/L sucrose. **d** Anthocyanin formation in rye shoots in MS medium supplemented with 1 mg/L 2,4-D + 1 mg/L TDZ plus 30 g/L sucrose. **e** Roots of plantlets in MS medium supplemented with 1 mg/L 2,4-D + 1 mg/L TDZ plus 30 g/L sucrose. **f** Acclimation of rye plantlets after transferring into soil

Fifty-eight plants produced from leaf base segments of rye were transferred to soil. Forty-seven plants were survived post acclimation with 81% success rate and monitored until mature stage. Based on observations, there was not any morphological differences between control plants (normal germinated plants through seeds) and in vitro regenerated plants. In addition, no albino and morphologically aberrant plants were observed (Fig. 2).

Shoot formation is a crucial step towards obtaining successful plant regeneration in in vitro culture. According to overall results, MS medium supplemented with 30 g/L sucrose and 2 mg/L 2,4-D + 1 mg/L TDZ was found as the best medium for shoot formation.

## Discussion

The fact that existence of a basal meristem cause competence of dedifferentiated cells making leaf base segments an excellent system in cereals (Wernicke and Brettell 1980, 1982). The regeneration potential of leaf base segments can also be used to produce transgenic plants as has been achieved in oats (Matsuda et al. 1998). In this study, efforts have been focused to gaining high regeneration proficiency from rye leaf basal segment. Acceptable amounts of somatic embryos were produced by only first leaf base segments, and calli derived from the first basal segment were the most effective for plant regeneration. Similar findings have been reported for other cereals such as wheat (Haliloglu 2006; Rajyalakshmi et al. 1991), barley (Becher et al. 1992) and oat (Chen et al. 1995; Gless et al. 1998). Whereas, second segment did not produce any callus nor produce any plant. This result was in accordance with an earlier idea that a decreasing gradient of response of callus formation from the base to the apex exists in leaves of cereals (Chen et al. 1995; Rajyalakshmi et al. 1991). Some researchers tried to explain the presence of such gradient due to orientation of the cells in the cell cycle, from apex to leaf base (Dolezelova et al. 1992). It is well acknowledged that plant growth regulators has critical role in in vitro regeneration (D’Onofrio and Morini 2003, 2005). Our results also pointed to that the presence of 2,4-D was important for callus induction and somatic embryo formation from leaf base segments of rye. It has been reported for cereals (Bhaskaran and Smith 1990; Gaspar et al. 1996) that use of cytokinins in combination with auxins to induce somatic embryogenesis in callus cultures. Conversely, cytokinin in callus initiation medium decreased the regeneration rate in the presented study. Some researchers also reported similar results in wheat (Wernicke and Milkovits 1984; Haliloglu 2006) and oat (Gless et al. 1998). Regeneration efficiency of the calli derived from leaf segment of rye is similar to that reported from mature and immature embryos (Eapen and Rao 1981; Rakoczy-Trojanowska and Malepszy 1993). In vitro regeneration protocol developed in this study also lacks season dependency as seedlings, the explant source, are available throughout the year.

Production of shoots from explant tissues often occurs via a callus phase and 2,4-D is one of the most potent auxins for induction of embryogenic callus. Further maturation of the embryos and plant development generally requires a culture medium containing cytokinins and a low concentration or no auxins. The plant growth regulator, TDZ, with both cytokinin- and auxin-like properties has been reported to promote plant regeneration in a number of species by stimulating production of axillary and/or adventitious shoots or somatic embryos (Huetteman and Preece 1993; Murthy and Saxena 1998). In cereals, TDZ-containing media have been used to promote shoot induction from wheat (Shan et al. 2000; Yu et al. 1999), barley (Ganeshan et al. 2006; Shan et al. 2000) and rice (Azria and Bhalla 2000; Tian et al. 1994) calluses. Gupta and Conger (1998) used a combination of TDZ and 2,4-D in the medium to induce multiple shoots directly from intact seedlings of switchgrass, *Panicum virgatum*. Nodal explants of *Bambusa edulis* have also been induced to produce multiple shoots in response to TDZ (Lin and Chang 1998). Similarly, for our study, calluses induced from leaf base segments produced shoots on a TDZ-containing medium.

In this study, we developed a protocol that is independent of season since seedlings as explant source, are available throughout the year. The use of leaf base segments for rye shoot regeneration eliminates the need for immature explant material, and consequently, growth of donor plants. Thus, the simplicity and rapid production of shoots from the leaf base segments could favor its use over the alternative explant sources. Besides being an efficient regeneration system, the leaf base system is relatively cost effective in terms of growth chamber and greenhouse space as it has no requirement for resources generally needed for growing donor plants. In addition, the rigorous growth conditions to maintain an optimal physiological state of donor plant material for maximal tissue culture response is eliminated. For these reasons, we anticipate that the leaf base system for rye could gain widespread use once genetic transformation has been demonstrated.

## Conclusions

Rye is well recognized to be one of the most recalcitrant cereals in terms of in vitro plant regeneration ability. This study presents a simple and fast regeneration system from leaf-base segment explant of rye. Basal media, carbohydrate source, combination of plant growth regulators and the leaf segment locations were evaluated for callus and shoot formation. The highest callus formation (10.39%) and shoot formation (4.53%) were achieved from first basal segments 3–4 days old seedlings. MS medium supplemented with 30 g/L sucrose and 2 mg/L 2,4-D + 1 mg/L TDZ was the best medium for shoot formation (18.75%) in first leaf base segment culture. The results indicate that this regeneration system can be used for molecular breeding programs of rye breeding such as genetic transformation and construction of mapping populations.
